# The STHLM3-model, Risk-based Prostate Cancer Testing Identifies Men at High Risk Without Inducing Negative Psychosocial Effects

**DOI:** 10.1016/j.euros.2020.12.010

**Published:** 2021-01-07

**Authors:** Marie Koitsalu, Martin Eklund, Jan Adolfsson, Mirjam A.G. Sprangers, Henrik Grönberg, Yvonne Brandberg

**Affiliations:** a Department of Oncology-Pathology, Karolinska Institutet, Stockholm, Sweden; b Department of Medical Epidemiology and Biostatistics, Karolinska Institutet, Stockholm, Sweden; c Department of Clinical Science, Intervention and Technology (CLINTEC), Karolinska Institutet, Stockholm, Sweden; d Department of Medical Psychology, Amsterdam University Medical Centers, Location AMC, Research Institute Cancer Center Amsterdam, University of Amsterdam, Amsterdam, The Netherlands

**Keywords:** Health behavior, Prostatic neoplasms, Risk-based screening, Worry

## Abstract

**Background:**

The new STHLM3 test, combining protein markers, genetic markers, and clinical data to assess a man’s prostate cancer (PCa) risk, has been investigated in Sweden within the frame of the STHLM3 trial.

**Objective:**

To assess whether the STHLM3 test influences men’s worry level, PCa knowledge, attitude, and health-related quality of life (HRQoL).

**Design, setting, and participants:**

Invitations with login to the web survey were mailed to 10 000 men, 50–69 yr of age, who were eligible for the STHLM3 trial. The survey was sent 3 mo *before* invitation to the STHLM3 trial (baseline) and 5 mo *after* STHLM3 (follow-up). At baseline, the men were unaware of the upcoming invitation to STHLM3. The survey covered the following: PCa-specific worry and perceived vulnerability, knowledge about PCa, attitude toward PCa testing and health behavior, and HRQoL

**Outcome measurements and statistical analysis:**

Survey scores were compared between baseline and follow-up by using the nonparametric Wilcoxon-signed rank tests for paired samples. Analysis of covariance was performed for PCa risk group comparisons.

**Results and limitations:**

A total of 994 men (10%) responded to our survey at baseline and follow-up, and were assessed as follows: low risk: 421 men; intermediate risk: 421 men; and high risk:152, of whom 59 were diagnosed with PCa after further investigation. In men assessed as having low and intermediate risk, level of worrying decreased at follow-up (*p* <  0.001), whereas no changes were observed in men at high risk. Moreover, no HRQoL changes were observed over time. The low response rate is the main limitation.

**Conclusions:**

We found that the STHLM3 model, a risk-based PCa test, showed no negative impact on the well-being of men.

**Patient summary:**

Since our results suggest that the risk-based screening as used in STHLM3 did not induce negative psychological effects on the participants, we can recommend this risk-based approach for population-based prostate cancer screening.

## Take Home Message

Performing a risk-based prostate cancer test did not appear to induce negative psychosocial effects. Being assessed as having low or intermediate risk was associated with decreased worry levels 5 mo after prostate cancer testing compared with the levels at baseline 3 mo before the test.

## 1 Introduction

Today, there is insufficient evidence to support the benefits of population-based screening for prostate cancer (PCa) using prostate-specific antigen (PSA). The harms associated with PSA screening have been considered to outweigh its benefits [1], [2]. Thus, no regulatory body has so far recommended PSA testing due to the potential concerns regarding unnecessary biopsies and overdiagnosis [2], [3]. Implementation of risk-based PCa screening has been proposed to reduce the harms of PSA screening [4]. Several studies have reported that tailoring screening to an individual’s risk level might improve the efficiency of the screening program and reduce its adverse consequences [5], [6], [7], [8]. However, little is known about how men would experience such risk-based PCa testing. Particularly, there is lack of knowledge about how attendance to risk-based PCa testing is associated with men’s worry about PCa, knowledge about PCa, and attitude toward PCa testing and health behavior, as well as their health-related quality of life (HRQoL). Insight into these aspects is needed to support the decision-making process of whether such risk-based screening should be implemented at population level.

We conducted a study among men 3 mo prior to and 5 mo following such risk-based testing for PCa. The aim of this study was to investigate changes from baseline to follow-up in PCa-specific worry and perceived vulnerability, knowledge about PCa, attitude toward PCa testing and health behavior, and HRQoL compared with the attributed risk groups.

## 2 Patients and methods

### 2.1 Study setting

The study was embedded in the STHLM3 trial, a population-based diagnostic study evaluating a blood-based PCa test predicting the risk for aggressive PCa [8]. The STHLM3 model uses a combination of plasma protein biomarkers, genetic polymorphisms, and clinical variables in order to investigate whether that model would identify men with PCa more effectively than PSA testing. The results of the study showed that the STHLM3 model reduced the rate of unnecessary biopsies significantly, without compromising the identification of high-risk PCa [8]. Participants in STHLM3 were randomly selected by date of birth from the Swedish Population Registry maintained by the Swedish Tax Agency. Men aged 50–69 yr were invited. Those who chose to participate in the STHLM3-study visited one of the 67 laboratories in Stockholm collaborating with STHLM3, in order to provide blood samples for the PCa risk assessment. A few weeks later, the participants received a response letter unveiling their test results and informing them as to which PCa risk level they belonged to. In accordance with their PCa risk assessment, the letter provided one of the following three recommendations: (1) “low risk” with the recommendation to perform a new test in 6 yr, (2) “normal risk” with the recommendation to have a new test in 2 yr, or (3) “increased risk” of PCa with the recommendation to consult a urologist for further examination and prostate biopsy.

### 2.2 Study design and population

We performed a longitudinal study inviting a total of 10 000 men. That cohort was due to be invited to STHLM3 during the month of April 2014. Men from Stockholm, Sweden, aged 50–69 yr and irrespective of any comorbidity except for PCa, were randomly selected for STHLM3 by date of birth. They were invited via mail to respond to a set of web-based questionnaires in January 2014, that is, the baseline assessment. At that time, these men were not aware of STHLM3. In April 2014, they were invited to participate in STHLM3 and thus undergo a PCa risk assessment. The same web-based questionnaires were then administered in September 2014, that is, the follow-up assessment, 5 mo after having undergone the PCa risk assessment and when they have been informed about the results. The letter, similar at baseline and follow-up, contained information about the questionnaire study and a login to the web survey. No reminders were sent. The inclusion criteria for this study were as follows: having responded to our survey both at baseline *and* at follow-up, having been tested within the STHLM3 study, and if assessed to have high risk for PCa, having performed a biopsy before follow-up. The study population was divided into four groups based on the results of the testing and further examinations: (1) “low risk,” (2) “intermediate risk,” (3) “high risk,” and (4) “PCa diagnosis.”

### 2.3 Study variables

The following questionnaires were used at both assessments (Supplementary material): PCa worry and perceived vulnerability [9], [10], [11], PCa knowledge [12], Attitude toward PCa testing and health behavior [13], and the EORTC QLQ-C30 for HRQoL [14]. All questionnaires were translated into Swedish by a certified translator and adapted to a web-based format. All original instruments had been used in previous international PCa testing studies [10], [11], [12], [15]. More information about the questionnaires can be found in a study published in 2018 [16].

### 2.4 Ethical approval

Ethical approval was obtained from the Regional Ethical Review Board in Stockholm (Dnr 2012/572-31/1, addition approved May 27, 2013). As stipulated in the invitation letter, completion of the survey was interpreted as informed consent to participate.

### 2.5 Statistical methods

#### 2.5.1 Preparation of item scores

The response options of the questions about PCa worry were dichotomized into “not at all worried” versus “some level of worry”, and those for the questions pertaining to perceived vulnerability were dichotomized into “none or small risk” versus “moderate to very high risk or do not know,” as well as “very or somewhat low” versus “moderate to very high or do not know” and “much less or less” versus “same-much more or do not know.” The respondents who responded ‘do not know’ for one of the three worry questions were omitted from the analysis (Q1: four men; Q2: five men; and Q3: seven men). Men who stated having previously been diagnosed with PCa were automatically (as a construct of the web-based format of the survey) not given the opportunity to respond to the worry questionnaire and taken to the following set of questions regarding knowledge. The answer to a previous PCa diagnosis was self-reported and apparently wrong, or the men would not have been able to perform prostate cancer testing (PCT). This led to 63 missing responses for the worry questionnaire (low risk: 23 men; intermediate risk: 14 men; high risk: 11 men; and PCa diagnosis: 15 men). We assumed that the missing data are missing completely at random and therefore decided to include the analysis. Response options for the knowledge questionnaire were dichotomized into right answer versus wrong answer/do not know.

Summary scores were produced for each scale in the questionnaire “Attitudes towards PCa testing and health behavior.” This was performed if half or more of the responses in the scale did not include the response option “do not know.” In addition, due to a technical error during data collection, the subscales (A) and (B) were missing at follow-up and could not be included in the analysis.

For the EORTC QLQ-C30 questionnaire, all scales were linearly transformed, ranging from 0 to 100. The “nausea and vomiting” scale as well as the single items, with the exception of “pain,” is not reported in this paper, as this was not considered pertinent to this study.

#### 2.5.2 Statistical analysis

Descriptive statistics were used to present the study sample.

For each of the four groups, questionnaire scores were compared between baseline and follow-up by using Wilcoxon-signed rank test for paired samples. Analysis of covariance, controlling for scores at baseline, was performed for group comparisons. We performed a large number of statistical tests. Since many of the tests are strongly correlated, it is challenging to formally control the false discovery rate. Therefore, for transparency and simplicity, we chose to use a 1% significance level for declaring statistical significance (instead of the standard 5% level) to reduce the number of false positive results.

## 3 Results

Out of the 1980 men responding to our questionnaire at baseline, 1347 underwent PCT in April 2014 and 1003 of those men responded at follow-up. Nine men were omitted as they responded at follow-up *before* having performed the biopsy. Thus, 994 men were included in the study. Among them, 421 men (42%) were identified to have “low risk,” 421 (42%) to have “intermediate risk,” and 152 (16%) to have “high risk.” Among men in the high-risk group, 59 (6%) received a confirmed PCa diagnosis after further investigation and were denoted as the “PCa diagnosis group,” whereas the remaining 93 men (10%) represented the “high-risk group.” The distribution of their age and education levels is presented in Table 1.

**Table 1:** Characteristics of participants (N = 994)

	Low risk		Intermediate risk		High risk		PCa diagnosis	
	*N*	%	*N*	%	*N*	%	*N*	%
Age (yr) a								
50–54	98	23	65	15	5	5	4	7
55–59	114	27	80	19	13	14	11	19
60–64	88	21	120	29	20	22	15	25
65–69	108	26	144	34	50	54	26	44
70+	11	3	12	3	5	5	3	5
Education a								
Elementary school	33	9	34	9	8	9	6	11
Upper secondary school	127	33	125	32	20	23	13	23
University	161	42	171	43	40	46	23	41
Other	64	17	64	16	19	22	14	25
PSA								
Before STHLM3 in 2014	261	71	268	72	95	73		
First time with STHLM3	108	29	102	28	35	27		

PCa = prostate cancer; PSA = prostate-specific antigen.

a Missing because self-reported.

### 3.1 Worry and perceived vulnerability to PCa

Statistically significant changes were observed in the low-risk group for all variables, indicating less worry and perceived vulnerability to PCa at follow-up (*p* <  0.001; Table 2). Similar results were found for the intermediate-risk group, with the exception of one item: “what do you think is your risk of getting PCa?”, where no significant change was observed. In the high-risk group, only one significant change was found—a higher proportion indicated a “very low” or “low” likelihood of developing cancer in the next 5 yr compared with baseline. A higher proportion in the “PCa diagnosis group” reported that PCa worry affected their daily life at follow-up compared with baseline, but no other significant changes were noted in that group.

**Table 2:** Comparison, by risk level, of men's worry and perceived vulnerability to PCa at baseline and follow-up^a^

	Low risk			Intermediate risk			High risk			PCa diagnosis		
	Baseline	Follow-up	*p* value b	Baseline	Follow-up	*p* value b	Baseline	Follow-up	*p* value b	Baseline	Follow-up	*p* value b
	*N* (%)	*N* (%)		*N* (%)	*N* (%)		*N* (%)	*N* (%)		*N* (%)	*N* (%)	
*Worry scale*												
1. How much do you worry about PCa?			<0.001			<0.001			0.1			0.4
Not at all	71 (18)	116 (29)		54 (13)	88(22)		12 (15)	7 (9)		8 (18)	5 (11)	
Some degree of worrying	324 (82)	279 (71)		352 (87)	318 (78)		70 (85)	75 (91)		36 (82)	39 (89)	
2. How much of a problem is PCa worry?			<0.001			<0.001			0.2			0.1
Not at all	215 (54)	287 (72)		207 (51)	259 (64)		34 (43)	40 (50)		23 (52)	16 (36)	
Some degree of worrying	181 (46)	109 (28)		199 (49)	147 (36)		46 (57)	40 (50)		21 (48)	28 (64)	
3. How much is your daily life affected by PCa worry?			<0.001			<0.001			0.8			<0.001
Not at all	325 (82)	352 (89)		326 (80)	353 (87)		67 (83)	68 (84)		37 (86)	24 (56)	
Some degree of worrying	70 (18)	43 (11)		79 (20)	52 (13)		14 (17)	13 (16)		6 (14)	19 (44)	
*Perceived vulnerability*												
4. What do you think is your risk of getting PCa?			<0.001			0.02			0.5			0.1
None or small	118 (30)	274 (69)		129 (32)	152 (37)		23 (28)	26 (32)		15 (34)	9 (20)	
Moderate-very high/do not know	280 (70)	124 (31)		278 (68)	255 (63)		59 (72)	56 (68)		29 (66)	35 (80)	
5. How likely do you think it is that you will develop PCa in the next 5 yr?			<0.001			<0.001			0.01			0.04
Very or somewhat low	264 (66)	345 (87)		285 (70)	334 (82)		52 (63)	63 (77)		24 (55)	15 (34)	
Moderate-very high/do not know	134 (34)	53 (13)		122 (30)	73 (18)		30 (37)	19 (23)		20 (45)	29 (66)	
6. Do you think you are more or less likely to get PCa?^c^			<0.001			<0.001			0.6			0.5
Much less or less	78 (20)	192 (48)		59 (15)	84 (21)		15 (18)	13 (16)		8 (18)	6 (14)	
Same-much more/do not know	320 (80)	206 (52)		348 (85)	323 (79)		67 (82)	69 (84)		36 (82)	38 (86)	

PCa = prostate cancer.

a 63 men missing due to technical error (low risk: 23 men; intermediate risk: 14 men; high risk: 11 men; PCa diagnosis: 15 men).

b Paired Wilcoxon signed rank test.

c Complete wording of question: In comparison with other men of your age and background, do you think you are more or less likely to get PCa?

### 3.2 PCa knowledge

Overall, apart from the first question where 75–90% of the respondents gave the correct answer, the vast majority answered incorrectly for the other questions, with proportions at baseline for the correct answer between 27% and 61% and at follow-up between 27% and 78% (Table 3). Group comparisons, controlling for baseline, were significant (*p* <  0.01) for “Q3,” “Q4,” and “Q5” when comparing the low-risk with the high-risk group, as well as when comparing the intermediate-risk with the high-risk group (not shown in the table), suggesting that men in the high-risk group had learned more over time than men in the low- and intermediate-risk group.

**Table 3:** Comparison, by risk level, of men's knowledge of PCa at baseline and follow-up

	Low risk			Intermediate risk			High risk			PCa diagnosis		
	Baseline	Follow-up	*p* value a	Baseline	Follow-up	*p* value a	Baseline	Follow-up	*p* value a	Baseline	Follow-up	*p* value a
	*N* (%)	*N* (%)		*N* (%)	*N* (%)		*N* (%)	*N* (%)		*N* (%)	*N* (%)	
1. How many men with early-stage PCa do you think will die of the disease?			0.08			0.002			0.2			0.01
Most will not b	344 (82)	359 (85)		348 (83)	372 (88)		79 (85)	85 (91)		45 (76)	53 (90)	
Wrong answer/do not know	77 (18)	62 (15)		73 (17)	49 (12)		14 (15)	8 (9)		14 (24)	6 (10)	
2. Does active treatment for early-stage PCa extend life?			0.08			0.05			0.7			<0.001
Pretty sure it can b	189 (45)	168 (40)		178 (42)	155(37)		38 (41)	40 (43)		36 (61)	16 (27)	
Wrong answer/do not now	232 (55)	253 (60)		243 (58)	266 (63)		55 (59)	53 (57)		23 (39)	43 (73)	
3. How many men with elevated PSA levels do you think have PCa?			<0.001			0.002			<0.001			0.2
Most do not b	114 (27)	161 (38)		117 (28)	150 (36)		34 (37)	59 (63)		22 (37)	28 (47)	
Wrong answer/do not know	307 (73)	260 (62)		304 (72)	271 (64)		59 (63)	34 (37)		37 (63)	31 (53)	
4. Do you think an infection or inflammation of the prostate can elevate PSA levels?			0.3			<0.001			0.001			0.1
Yes b	182 (43)	194 (46)		153 (36)	200 (48)		45 (48)	62 (67)		25 (42)	31 (53)	
No/do not know	239 (57)	227 (54)		267 (64)	220 (52)		48 (52)	31 (33)		34 (58)	28 (47)	
5. Do you think a large prostate can elevate PSA levels?			0.7			0.6			<0.001			0.2
Yes b	204 (48)	209 (50)		208 (50)	214 (51)		51 (55)	73 (78)		33 (56)	39 (66)	
No/do not know	217(52)	212 (50)		212 (50)	206 (49)		42 (45)	20 (22)		26 (44)	20 (34)	
6. Do you think a prostate biopsy can miss some cancer?			0.9			0.9			0.3			0.8
Yes b	162 (38)	164 (39)		185 (44)	185 (44)		53 (57)	47 (51)		29 (49)	30 (51)	
No/do not know	259 (62)	257 (61)		235 (56)	235(56)		40 (43)	46 (49)		30 (51)	29 (49)	

PCa = prostate cancer; PSA = prostate-specific antigen.

a Paired Wilcoxon signed rank test.

b Denotes correct answer.

### 3.3 Attitude and health behavior

Both the low-risk and the intermediate-risk group scored statistically significantly lower on “external influences” and “general health” at follow-up compared with baseline. No other statistically significant changes were found. In the high-risk and he PCa diagnosis groups, “barriers” increased, but no other significant changes were found between baseline and follow-up for any of the remaining variables (Table 4).

**Table 4:** Comparison, by risk level, of men's attitude and health behavior mean scores at baseline and follow-up

Scales	Risk level	Baseline		Follow-up			Between-group difference at follow-up a	
		*N*^b^	Mean (SD)	*N*^b^	Mean (SD)	*p* value c	Adj. Mean diff. (99% CI)	*p* value
Barriers d								
	Low risk	403	18.4 (5.8)	394	18.3 (5.2)	0.6	–	
	Intermediate risk	406	18.4 (5.4)	413	18.2 (5.1)	0.3	–0.2 (–1.0 to 0.7)	0.6
	High risk	86	19.1 (5.5)	87	21.5 (5.2)	<0.001	2.7 (1.3 to 4.1)	<0.001
	PCa diagnosis	58	18.9 (5.7)	44	21.3 (6.9)	0.006	2.8 (0.9 to 4.6)	<0.001
Intention e								
	Low risk	391	1.3 (0.7)	377	1.2 (0.7)	0.04	–	
	Intermediate risk	402	1.3 (0.8)	401	1.2 (0.7)	0.03	–0.03 (–0.15 to 0.09)	0.6
	High risk	83	1.3 (0.6)	85	1.1 (0.6)	0.03	–0.08 (–0.3 to 0.1)	0.3
	PCa diagnosis	52	1.2 (0.7)	44	1.1 (0.6)	0.4	–0.09 (–0.38 to 0.19)	0.4
External influences f								
	Low risk	400	9.6 (3.6)	394	8.7 (3.9)	<0.001	–	
	Intermediate risk	404	9.3 (3.6)	409	8.4 (3.8)	<0.001	–0.2 (–0.8 to 0.4)	0.5
	High risk	85	9.1 (3.4)	87	9.3 (3.9)	0.6	0.7 (–0.4 to 1.8)	0.1
	PCa diagnosis	57	9.2 (4.0)	43	8.3 (3.8)	0.3	0.07 (–1.4 to 1.5)	0.9
General health g								
	Low risk	410	7.9 (1.7)	399	7.7 (1.8)	0.01	–	
	Intermediate risk	411	7.9 (1.8)	414	7.7 (1.8)	0.005	–0.02 (–0.3 to 0.3)	0.8
	High risk	87	8.0 (1.6)	87	8.0 (1.8)	0.9	0.3 (–0.2 to 0.8)	0.1
	PCa diagnosis	59	7.7 (1.9)	44	8.0 (1.5)	0.3	0.4 (–0.2 to 1.04)	0.1

Adj. mean diff. = adjusted mean difference; ANCOVA = analysis of covariance; CI = confidence interval; PCa = prostate cancer; SD = standard deviation.

a Adjusted mean difference at follow-up of pairwise comparisons with low-risk group by using ANCOVA, adjusted for baseline.

b N varies because participants who had responded "do not know" to more than half of the response items for a specific scale were excluded.

c Paired Wilcoxon signed rank test.

d Range 1–50; ten items.

e Range 1– 5; one item.

f Range 1–15; three items.

g Range 1–10; two items.

Table 4 also presents the results of the comparisons between intermediate risk, high risk, PCa diagnosis, and low-risk groups. At follow-up, no differences were found between the low-risk group and the other groups, with one exception. The high-risk and PCa diagnosis groups scored statistically significantly higher on “barriers to screening” than the low-risk group. This difference (*p* <  0.001) was also found when comparing the intermediate-risk group with the high-risk and PCa diagnosis groups (not shown in the table).

### 3.4 Health-related quality of life

No statistically significant changes between baseline and follow-up were found for HRQoL in any of the groups, with one exception. Emotional functioning improved over time in the intermediate-risk group (Table 5).

**Table 5:** Comparison, by risk level, of men's EORTC QLQ-C30 mean scores at baseline and follow-up

Scales	Risk level	Baseline		Follow-up			Between-group difference at follow-up a	
		*N*	Mean (SD)	*N*	Mean (SD)	*p* value b	Adj. Mean diff. (99% CI)	*p* value
Global health status c								
	Low risk	421	81 (19)	421	82 (19)	0.4	–	
	Intermediate risk	421	84 (16)	421	85 (14)	0.03	1.5 (–0.7 to 3.7)	0.1
	High risk	93	82 (17)	93	85 (14)	0.03	2.4 (–1.2 to 6.0)	0.1
	PCa diagnosis	59	82 (15)	59	84 (13)	0.2	1.4 (–3.0 to 5.7)	0.4
Physical functioning c								
	Low risk	420	96 (11)	420	96 (10)	0.4	–	
	Intermediate risk	421	97 (9)	421	97 (8)	0.4	–0.1 (–1.1 to 0.9)	0.8
	High risk	93	98 (7)	93	98 (7)	0.4	0.5 (–1.1 to 2.1)	0.4
	PCa diagnosis	59	98 (6)	59	99 (4)	0.05	1.6 (–0.4 to 3.6)	0.04
Role functioning c								
	Low risk	421	94 (17)	419	94 (18)	0.6	–	
	Intermediate risk	421	96 (14)	420	96 (12)	0.9	1.3 (–0.9 to 3.5)	0.1
	High risk	93	94 (14)	93	95 (12)	0.4	1.5 (–2.1 to 5.2)	0.3
	PCa diagnosis	59	92 (19)	59	97 (11)	0.02	4.0 (–0.5 to 8.4)	0.03
Emotional functioning c								
	Low risk	421	88 (16)	419	89 (16)	0.4	–	
	Intermediate risk	420	89 (16)	421	91 (14)	0.006	1.4 (–0.7 to 3.6)	0.08
	High risk	93	90 (15)	93	91 (16)	0.7	1.2 (–2.3 to 4.8)	0.4
	PCa diagnosis	59	91 (13)	59	88 (14)	0.09	1.2 (–2.3 to 4.8)	0.1
Cognitive functioning c								
	Low risk	421	91 (14)	420	90 (15)	0.3	–	
	Intermediate risk	420	92 (14)	421	92 (13)	0.6	1.1 (–0.8 to 3.1)	0.1
	High risk	93	90 (15)	93	90 (14)	0.9	–0.1 (–3.4 to 3.2)	0.9
	PCa diagnosis	59	92 (14)	59	89 (14)	0.1	–0.1 (–3.4 to 3.2)	0.3
Social functioning c								
	Low risk	420	94 (16)	417	95 (14)	0.2	–	
	Intermediate risk	420	94 (15)	420	95 (14)	0.2	0.4 (–1.7 to 2.6)	0.6
	High risk	93	92 (17)	93	94 (14)	0.2	0.0 (–3.6 to 3.6)	0.9
	PCa diagnosis	59	95 (11)		94 (13)	0.5	–1.3 (–5.7 to 3.1)	0.4
Pain d								
	Low risk	421	11 (20)	420	11 (19)	0.6	–	
	Intermediate risk	420	10 (18)	421	9 (18)	0.7	–0.6 (–3.2 to 2.0)	0.5
	High risk	93	10 (18)	93	8 (14)	0.2	–2.1 (–6.4 to 2.3)	0.2
	PCa diagnosis	59	12 (21)	59	6 (12)	0.04	–5.4 (–10.7 to –0.12)	0.009
Fatigue d								
	Low risk	421	15 (18)	420	14 (19)	0.8	–	
	Intermediate risk	420	13 (17)	421	12 (15)	0.09	–1.4 (–3.7 to 0.8)	0.1
	High risk	93	13 (16)	93	12 (15)	0.3	–1.9 (–5.7 to 1.9)	0.2
	PCa diagnosis	59	14 (15)	59	10 (13)	0.02	–4.0 (–8.6 to 0.6)	0.02

Adj. Mean diff. = adjusted mean difference; ANCOVA = analysis of covariance; CI = confidence interval; PCa = prostate cancer; SD = standard deviation.

a Adjusted mean difference at follow-up of pairwise comparisons with low-risk group by using ANCOVA, adjusted for baseline.

b Paired Wilcoxon signed rank test.

c Range 0–100; high levels represent high levels of functioning and quality of life.

d Range 0–100; high levels represent high levels of problems.

At follow-up, no statistically significant differences were found between the low-risk group and the other groups, with the exception of pain (lower score in the PCa diagnosis group).

## 4 Discussion

The study revealed no negative changes in worry for PCa, HRQoL, or health behavior between the baseline assessments, 3 mo before participation in risk-based PCa testing, and follow-up 5 mo after the risk assessment in any of the groups. Between baseline and follow-up, the number of men worrying about PCa decreased for those who were assessed as having low and intermediate risk, and did not increase for those who were assessed as having high risk for PCa. The findings are reassuring for risk-based screening programs, where a negative psychological impact could adversely influence screening intention or reattendance rates. Our results are in line with other studies [17], [18] reporting no increase in anxiety after familial risk assessment for common diseases.

Interestingly, the proportion of men in the high-risk group who reported that their risk of developing PCa in the next 5 yr was “very or somewhat low” increased between baseline and follow-up. One explanation could be that at the time of follow-up, these men had undergone additional testing and found out that they were PCa free, whereas the men in the high-risk group who subsequently received a confirmed diagnosis comprised the PCa diagnosis group.

As for the men in the PCa diagnosis group, the only statistically significant finding concerns the question about how much their daily life is affected by PCa worry. At follow-up, these men have recently been diagnosed with PCa, and thus the increase in worrying was expected.

Overall, apart from the first question concerning PCa knowledge, the vast majority answered incorrectly for the other questions. Even when significant changes were observed within the low- and intermediate-risk groups, these improvements still did not represent a majority of correct answers at follow-up. Group comparisons suggested that men in the high-risk group learned more over time than men in the low- and intermediate-risk groups. This concurs with the idea of searching information when diagnosed with a disease [19]. Our study showed that more information and knowledge need to be relayed when introducing PCa screening, in order for men to make an informed choice when undergoing screening.

As for attitude and health behaviors, the low- and intermediate-risk groups reported a small decrease in how strongly they agreed that external influences would impact their PCa testing decision-making. They also reported a small decrease in how strongly they agreed about the importance of maintaining good health and searching for new information to improve it. Both these results reflect a sign of decreased worry concerning PCa and the general health of men following their assessment as low- and intermediate-risk groups. The high-risk and PCa diagnosis groups reported an increase in perceived barriers to PCa testing, perhaps due to having had to undergo further testing, as opposed to the low- and intermediate-risk groups.

Surprisingly, men diagnosed with PCa showed lower levels of pain at follow-up than at baseline. We cannot rule out that this is a chance finding.

Our study has strengths and limitations. The selection of participants may have been biased toward those interested or concerned about PCa risk, which may have caused a selection bias. Out of the 1980 men who responded at baseline, 70% proceeded to undergo PCT. This cohort was compared with the 30% of men who responded at baseline but did not undergo PCT, as described by Koitsalu et al [16] in 2018. This article showed, among others, that men declining PCT are slightly less worried than participants and perceive fewer benefits, thus pointing to a selection bias between participants and decliners at baseline. The percentages of men across risk groups at follow-up equaled those at baseline (low risk: 41%; intermediate risk: 42%; and high risk: 17%). Hence, there is no evidence that men who declined participation at follow-up did so due to their risk allocation. Moreover, the risk distribution in our study was highly comparable with the one observed in the larger STHLM3 trial [8], where low-, intermediate-, and high-risk groups represented 44%, 40%, and 16%, respectively. Unfortunately, we do not have data to examine possible differences in psychosocial profiles between our cohort and the STHLM3 cohort. Although selective attrition was not likely, the absolute response rate was 10%, which is low and limits the possibility to draw conclusions.

The original English questionnaires were translated into Swedish by a certified translation company, using a forward-backward translation procedure. Despite the professional approach, we cannot guarantee that the questionnaires are linguistically equivalent to the original ones.

We dichotomized the responses leading to loss of information in variation among individuals. However, for the purpose of the present study, we wanted to focus on the final message (worried or not, right or wrong, etc.) rather than on the variation in their responses. Furthermore, our dichotomizations were justified as, for the majority of the variables, the distributions were highly skewed toward one response category. Moreover, we performed a number of analyses at item level, which we considered warranted as we intended to gain insight into the item content conforming to the study aims. Since this led to a large number of statistical tests, the level of statistical significance were set at 1%.

We are aware that the questions in the worry questionnaire pertaining to perceived vulnerability to PCa were not suited for men who ended up with a PCa diagnosis and may have been confusing for them. However, we decided to present all the results in Table 2 to facilitate comparisons of the groups at baseline, since at baseline they do not yet know which risk group they belong to. We intentionally did not interpret those questions for this group at follow-up.

The strengths of our study are that the sample was population based and relatively large. Another strength is that standardized questionnaires were used, which had been employed in previous studies of PCa screening. The longitudinal design, using well-defined assessment points, is also a strength. Moreover, since our baseline was measured before any involvement in PCa risk assessment, it can be considered a true baseline. Finally, the risk groups are based on biological data and include a confirmed PCa group.

## 5 Conclusions

We conclude that the PCa risk testing used in STHLM3 did not induce worry or decrease HRQoL. The study also pointed out the importance of providing information to men seeking PCa testing, as knowledge about PCa in the population seems to be lacking. Hence, from a psychological point of view, there may not be any adverse effects for this risk-based screening for PCa in the general population.

  ***Author contributions*:** Marie Koitsalu had full access to all the data in the study and takes responsibility for the integrity of the data and the accuracy of the data analysis.

  *Study concept and design*: Koitsalu, Brandberg, Sprangers, Grönberg.

*Acquisition of data*: Koitsalu, Grönberg, Brandberg.

*Analysis and interpretation of data*: Koitsalu, Eklund, Brandberg.

*Drafting of the manuscript*: Koitsalu, Brandberg.

*Critical revision of the manuscript for important intellectual content*: Koitsalu, Eklund, Adolfsson, Sprangers, Grönberg, Brandberg.

*Statistical analysis*: Koitsalu, Eklund.

*Obtaining funding*: Grönberg, Brandberg.

*Administrative, technical, or material support*: Grönberg, Brandberg.

*Supervision*: Brandberg.

*Other*: None.

  ***Financial disclosures:*** Marie Koitsalu certifies that all conflicts of interest, including specific financial interests and relationships and affiliations relevant to the subject matter or materials discussed in the manuscript (eg, employment/affiliation, grants or funding, consultancies, honoraria, stock ownership or options, expert testimony, royalties, or patents filed, received, or pending), are the following: Henrik Grönberg has five PCa diagnostic–related patents pending, and might receive royalties from sales related to these patents. Martin Eklund is named on four of these five patent applications.

  ***Funding/Support and role of the sponsor*:** This work was supported by the Cancer Risk Prediction Center (CRisP), a Linneus Centre (contract ID 70867901) financed by the Swedish Research Council (Vetenskapsrådet); and by the Swedish Cancer Society (Cancerfonden). The funders had no role in study design, data collection and analysis, preparation of the manuscript, or decision to publish the manuscript.

## CRediT authorship contribution statement

**Marie Koitsalu:** Conceptualization, Methodology, Validation, Formal analysis, Investigation, Data curation, Writing - original draft, Visualization. **Martin Eklund:** Software, Validation, Formal analysis, Data curation. **Jan Adolfsson:** Writing - review & editing. **Mirjam A.G. Sprangers:** Conceptualization, Methodology, Writing - review & editing, Supervision. **Henrik Grönberg:** Conceptualization, Resources, Project administration, Funding acquisition. **Yvonne Brandberg:** Conceptualization, Methodology, Investigation, Resources, Writing - original draft, Supervision, Project administration, Funding acquisition.
