# Supplementary material for: The STHLM3-model, Risk-based Prostate Cancer Testing Identifies Men at High Risk Without Inducing Negative Psychosocial Effects
Source: Eur Urol Open Sci. 2021 Jan 7;24:43–51. doi: 10.1016/j.euros.2020.12.010 (PMC8317863; doi:10.1016/j.euros.2020.12.010)
Supplement: Supplementary file 1 [file mmc1.docx]

Supplementary materials

English version of the Questionnaires:

Items and response options for the Prostate cancer specific worry and perceived vulnerability questionnaire:

*Worry scale*

1. How much do you worry about prostate cancer (PCa)?

Not at all, a little, a lot, very much, do not know

1. How much of a problem is PCa worry?

Not at all, a little, a lot, very much, do not know

1. How much is your daily life affected by PCa worry?

Not at all, a little, a lot, very much, do not know

*Perceived vulnerability*

1. What do you think is your risk of getting PCa?

None, small risk, moderate risk, high risk, very high risk, do not know

1. How likely do you think it is that you will develop PCa in the next 5 years?

Very unlikely, somewhat unlikely, quite likely, very likely, do not know

1. In comparison to other men of your age and background, do you think you are more or less likely to get PCa?

Much less, less, about the same, more, much more, do not know

Items and response options for the Knowledge questionnaire:

1. How many men with early-stage prostate cancer (PCa) do you think will die of the disease?

Most or all will, about half, most will not, do not know

1. Does active treatment for early-stage PCa extend life?

Very sure it can, pretty sure it can, not sure, pretty sure it cannot, do not know

1. How many men with elevated PSA levels do you think have PCa?

Most or all do, about half, most do not, do not know

1. Do you think an infection or inflammation of the prostate can elevate PSA levels?

Yes, no, do not know

1. Do you think a large prostate can elevate PSA levels?

Yes, no, do not know

1. Do you think a prostate biopsy can miss some cancers?

Yes, no, do not know

Scales and items for the Attitudes and Health behaviour questionnaire. Responses for all items range from 1 to 5 (strongly disagree, disagree, neither disagree nor agree, agree, strongly agree) (PCa: prostate cancer)

1. Perceived threat of developing prostate cancer (PCa) (2 items)

There is a high possibility that I will get PCa in my lifetime.

The older I get, the more likely I am to get PCa.

1. Perceived benefits of PCa testing (8 items)

Having a PCa test would give me peace of mind.

Having a PCa test would mean I wouldn’t worry as much about PCa.

Having a PCa test would allow finding PC early.

Having a PCa test would mean fewer men would die from PCa.

Having a PCa test would decrease my chance of dying from PCa.

I want to discover health problems early.

I believe that PCa testing can help protect my health.

Treatment for PCa is more successful the earlier it is detected.

1. Perceived barriers to PCa testing (10 items)

Having a PCa test would be an easy thing for me to do.

Having a PCa test would make me worry about PCa.

Having a PCa blood test is painful.

Having a PCa test would take too much time/be inconvenient.

I am worried that a PCa blood test will show that I have PCa.

PCa testing is not needed if you do not have symptoms.

I am afraid that I might need to have treatment if I have a PCa test that shows I have PCa.

I am worried that a prostate biopsy will show that I have PCa.

Having a prostate biopsy would be embarrassing.

Having a prostate biopsy would be painful.

1. Intentions to undergo PCa testing (1 item)

I do not intend to undergo a PCa blood test.

1. External influences on PCa testing decision making (3 items)

Knowing someone with PCa could influence my decision to have a PCa test.

Recent publicity about PCa could influence my decision to have a PCa test.

Talking to my doctor could influence my decision to have a PCa test.

1. General health (2 items)

Maintaining good health is extremely important for me.

I search for new information to improve my health.
